# Effect of small-vessel disease on cognitive trajectory after atrial fibrillation-related ischaemic stroke or  TIA

**DOI:** 10.1007/s00415-019-09256-6

**Published:** 2019-03-07

**Authors:** Gargi Banerjee, Edgar Chan, Gareth Ambler, Duncan Wilson, Lisa Cipolotti, Clare Shakeshaft, Hannah Cohen, Tarek Yousry, Gregory Y. H. Lip, Keith W. Muir, Martin M. Brown, Hans Rolf Jäger, David J. Werring, The CROMIS-2 collaborators, Rustam Al-Shahi Salman, Louise Shaw, Kirsty Harkness, Jane Sword, Azlisham Mohd Nor, Pankaj Sharma, Deborah Kelly, Frances Harrington, Marc Randall, Matthew Smith, Karim Mahawish, Abduelbaset Elmarim, Bernard Esisi, Claire Cullen, Arumug Nallasivam, Christopher Price, Adrian Barry, Christine Roffe, John Coyle, Ahamad Hassan, Caroline Lovelock, Jonathan Birns, David Cohen, L. Sekaran, Adrian Parry-Jones, Anthea Parry, David Hargroves, Harald Proschel, Prabel Datta, Khaled Darawil, Aravindakshan Manoj, Mathew Burn, Chris Patterson, Elio Giallombardo, Nigel Smyth, Syed Mansoor, Ijaz Anwar, Rachel Marsh, Sissi Ispoglou, Dinesh Chadha, Mathuri Prabhakaran, Sanjeevikumar Meenakishundaram, Janice O’Connell, Jon Scott, Vinodh Krishnamurthy, Prasanna Aghoram, Michael McCormick, Paul O’Mahony, Martin Cooper, Lillian Choy, Peter Wilkinson, Simon Leach, Sarah Caine, Ilse Burger, Gunaratam Gunathilagan, Paul Guyler, Hedley Emsley, Michelle Davis, Dulka Manawadu, Kath Pasco, Maam Mamun, Robert Luder, Mahmud Sajid, Ijaz Anwar, James Okwera, Julie Staals, Elizabeth Warburton, Kari Saastamoinen, Timothy England, Janet Putterill, Enrico Flossman, Michael Power, Krishna Dani, David Mangion, Appu Suman, John Corrigan, Enas Lawrence, Djamil Vahidassr

**Affiliations:** 1 Department of Brain Repair and Rehabilitation, Stroke Research Centre, UCL Queen Square Institute of Neurology and National Hospital for Neurology and Neurosurgery, Russell Square House, 10-12 Russell Square, London, WC1B 5EH UK; 2 Department of Neuropsychology, National Hospital for Neurology and Neurosurgery, Queen Square, London, UK; 3 Department of Statistical Science, University College London, Gower Street, London, UK; 4 New Zealand Brain Research Institute, Christchurch, New Zealand; 5 Haemostasis Research Unit, Department of Haematology, University College London, 51 Chenies Mews, London, UK; 6 Lysholm Department of Neuroradiology and the Neuroradiological Academic Unit, Department of Brain Repair and Rehabilitation, UCL Queen Square Institute of Neurology, Queen Square, London, UK; 7 Liverpool Centre for Cardiovascular Science, University of Liverpool and Liverpool Heart and Chest Hospital, Liverpool, UK; 8 Aalborg Thrombosis Research Unit, Department of Clinical Medicine, Aalborg University, Aalborg, Denmark; 9 Institute of Neuroscience and Psychology, University of Glasgow, Queen Elizabeth University Hospital, Glasgow, UK

**Keywords:** Atrial fibrillation, Brain ischaemia, Cerebral small-vessel disease, Cognitive impairment, Ischaemic stroke, Transient ischaemic attack (TIA)

## Abstract

Post-stroke dementia is common but has heterogenous mechanisms that are not fully understood, particularly in patients with atrial fibrillation (AF)-related ischaemic stroke or TIA. We investigated the relationship between MRI small-vessel disease markers (including a composite cerebral amyloid angiopathy, CAA, score) and cognitive trajectory over 12 months. We included patients from the CROMIS-2 AF study without pre-existing cognitive impairment and with Montreal Cognitive Assessment (MoCA) data. Cognitive impairment was defined as MoCA < 26. We defined “reverters” as patients with an “acute” MoCA (immediately after the index event) score < 26, who then improved by ≥ 2 points at 12 months. In our cohort (*n* = 114), 12-month MoCA improved overall relative to acute performance (mean difference 1.69 points, 95% CI 1.03–2.36, *p* < 0.00001). 12-month cognitive impairment was associated with increasing CAA score (per-point increase, adjusted OR 4.09, 95% CI 1.36–12.33, *p* = 0.012). Of those with abnormal acute MoCA score (*n* = 66), 59.1% (*n* = 39) were “reverters”. Non-reversion was associated with centrum semi-ovale perivascular spaces (per-grade increase, unadjusted OR 1.83, 95% CI 1.06–3.15, *p* = 0.03), cerebral microbleeds (unadjusted OR 10.86, 95% CI 1.22–96.34, *p* = 0.03), and (negatively) with multiple ischaemic lesions at baseline (unadjusted OR 0.11, 95% CI 0.02–0.90, *p* = 0.04), as well as composite small-vessel disease (per-point increase, unadjusted OR 2.91, 95% CI 1.23–6.88, *p* = 0.015) and CAA (per-point increase, unadjusted OR 6.71, 95% CI 2.10–21.50, *p* = 0.001) scores. In AF-related acute ischaemic stroke or TIA, cerebral small-vessel disease is associated both with cognitive performance at 12 months and failure to improve over this period.

## Introduction

Post-stroke dementia is common [30] but has heterogenous mechanisms that are not fully understood. Early post-stroke dementia (within 6 months) is associated with factors relating to brain resilience and the index stroke lesion, whereas delayed-onset post-stroke dementia is more associated with cerebral small-vessel diseases [23]. This appears to be the case for both ischaemic and haemorrhagic stroke; dementia after ischaemic intracerebral events (stroke or transient ischaemic attacks, TIA) is associated with white-matter hyperintensities, lacunes and cortical atrophy [1, 24], and markers of cerebral amyloid angiopathy (CAA) are associated with dementia after intracerebral haemorrhage [25]. The contribution of small-vessel disease to post-stroke dementia in patients with atrial fibrillation (AF)-related ischaemic stroke or TIA is unknown. AF is recognised as an independent risk factor for dementia [20]; proposed mechanisms include silent brain infarction from recurrent embolization, cerebral hypoperfusion, chronic inflammation and endothelial dysfunction, or progression of pre-existing cerebrovascular or neurodegenerative processes [31], but information on the imaging associations of post-stroke dementia in this context remains limited.

The natural history of post-stroke dementia is further complicated by the fact that cognitive performance immediately after a stroke might not be representative of later cognition, as performance might improve; this occurs both acutely, where the initial assessment might be influenced by delirium, but also over longer time periods [2, 10, 17, 19, 29]. The Montreal Cognitive Assessment (MoCA) appears to be a sensitive cognitive screen for identifying these changes [34], and there has been recent interest in identifying the characteristics of so-called “reverters”, who demonstrate improvements in their cognitive performance over time [27, 34, 38, 43].

We investigated cognitive trajectory in patients with AF-related ischaemic stroke or TIA. Our objectives were: (1) to describe the changes in MoCA that occur between acute (immediately post ischaemic event) and 12 month assessment, (2) to investigate the clinical and radiological associations of MoCA-defined cognitive impairment at 12 months, and (3) to describe the clinical and imaging profile of reverters and non-reverters.

## Materials and methods

### Patient selection

This is a subgroup analysis of the CROMIS-2 AF study, the protocol for which has been described [7, 41]. Briefly, this was a multi-centre prospective observational study of adults (aged 18 years or above) presenting with ischaemic cardioembolic stroke or TIA with non-valvular atrial fibrillation (confirmed by electrocardiography), who were eligible to start anticoagulation following their ischaemic event [7, 41]. Patients who could not have an MRI scan, had contraindications to anticoagulation, or had previously received therapeutic anticoagulation, were excluded [7, 41]. The study was approved by the National Research Ethics Service (IRAS reference 10/H0716/61), and written informed consent was obtained for each patient.

**Fig. 1:**
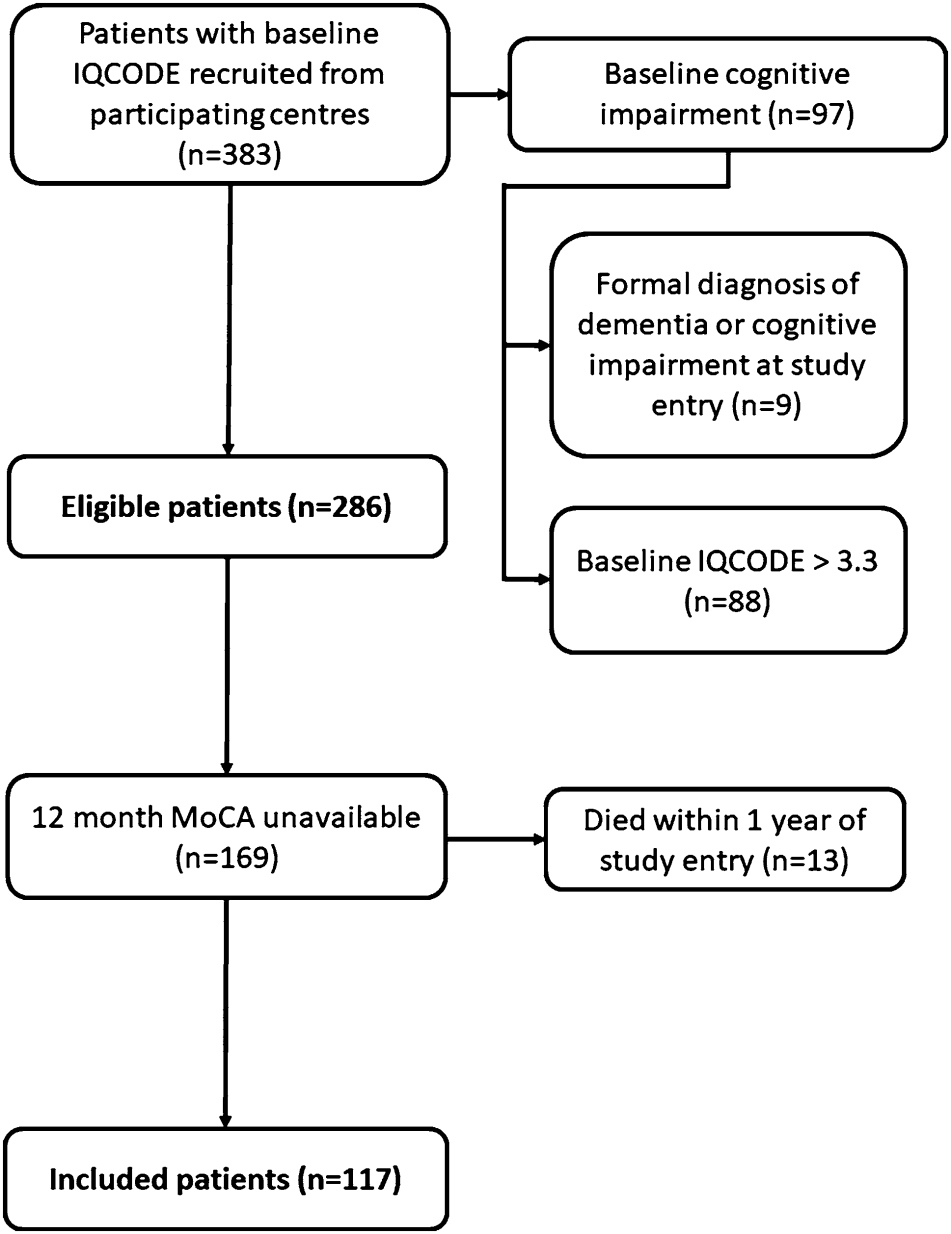
Description of study population

We excluded patients with known documented diagnosis of dementia or cognitive impairment, or 16-item IQCODE (Informant Questionnaire for Cognitive Decline in the Elderly) score > 3.3 [16] at study entry (Fig. 1). We compared characteristics of eligible (those with 12 month MoCA data) and excluded patients (those without) to assess selection bias.

### Cognitive measures

The “acute” MoCA was collected immediately after the index ischaemic event. All participating centres were invited to collect additional MoCA data at 12 months (“12 month MoCA”) following study entry; 20 centres agreed to contribute to this substudy. A MoCA score < 26 was used to define cognitive impairment [26]. “Reverters” were defined as patients with an acute MoCA score < 26, who demonstrated an improvement of ≥ 2 points at 12 months, and patients with an acute MoCA score < 26 who did not show this improvement were defined as “non-reverters”. These thresholds are based on definitions from previously published work [27, 34].

### Imaging

Imaging was undertaken locally at each study centre in accordance with a standardised protocol including axial T2, T2*-GRE, diffusion-weighted imaging, coronal T1 and FLAIR images [7]. Sequence parameters were specified for T2*-GRE [7]; the remaining sequences were obtained according to local protocols.

Neuroimaging analysis was carried out by two clinical research associates trained in rating structural markers of small-vessel disease and blinded to clinical details. Each structural marker was rated by a single rater, and all structural markers of cerebral small-vessel disease were rated in accordance with consensus criteria [40], and, where possible, the hemisphere contralateral to the acute stroke was preferentially counted.

Previous cortical infarcts were identified using T2 and FLAIR sequences and confirmed as non-acute through comparison with diffusion-weighted images (DWI). Lacunes were identified and counted on T2 and FLAIR sequences using definitions from the STRIVE criteria [40]. White-matter hyperintensities in deep (dWMH) and periventricular (pvWMH) distributions were rated on T2 and FLAIR sequences using the Fazekas scale [11, 12]. MRI-visible perivascular spaces in the basal ganglia (BG-PVS) and centrum semi-ovale (CSO-PVS) were rated on T2 and FLAIR sequences using a previously described validated visual rating scale [9, 22]. Medial temporal atrophy (MTA) was rated on T1 or FLAIR coronal images using the Scheltens visual scale [15]. Global cortical atrophy (GCA) was rated with the Pasquier scale [28] using axial T1, FLAIR or inverted T2 images. Cortical superficial siderosis (cSS) was identified on T2*-GRE sequences and classified as either focal, involving three or fewer sulci, or disseminated, involving four or more sulci [5]. Cerebral microbleeds (CMB) were rated using T2*-GRE sequences using the Microbleed Anatomical Rating Scale (MARS) [13]. Composite SVD [36, 37] and CAA [6] scores were determined using previously described scales.

Acute DWI lesions were defined as areas bright on the B1000 and dark on the corresponding ADC map; the side of the lesion, presence of single or multiple acute lesions, and evidence of cortical involvement were recorded. Evidence of haemorrhagic transformation was rated using the ECASS classification [32] using T2*-GRE sequences.

### Statistics

We compared baseline clinical, demographic and imaging findings in patients with and without MoCA-defined cognitive impairment at 12 months, and for reverters compared with non-reverters. For all continuous variables, data were reviewed for normality, and if normally distributed, the independent t-test was used. If variables were ordinal or not normally distributed, the non-parametric Mann–Whitney *U* test was used. Chi-squared or Fisher’s exact tests were used for categorical variables. Performances of acute and 12 month MoCA were compared using paired *t* tests (mean scores) or McNemar’s test (proportion impaired).

The results of univariate comparisons were used to identify variables for inclusion in multivariable logistic regression models; all variables with *p* < 0.20 were included in the adjusted analyses except for situations where variables both described the same phenomenon (for example, clinical history of previous ischaemic events and imaging evidence of a previous cortical infarct). Adjusted models considered only a single neuroimaging marker at a time. Given that these analyses were exploratory, we did not make an adjustment for multiple testing.

Statistical analysis was performed (GB) using Stata (Version 11.2).

## Results

### Participants

383 patients were recruited from the 20 sites participating in the MoCA substudy: 286 patients were eligible for this substudy, of whom 117 had 12 month cognitive data available (Fig. 1). The characteristics of those with 12 month MoCA data and those without (i.e. those excluded from this substudy) are shown in Table 1. Those included within the analysis had a mean age of 73.1 years; 45 (38.5%) were female, and the median NIHSS on admission was 3.5.

**Table 1:** Baseline characteristics of included and excluded patients

	Included (*n* = 117)	Excluded (*n* = 169)	*p* value
Age (years), mean (SD)	73.1 (9.1)	74.2 (10.7)	0.3757
Sex, female, *n* (%)	45 (38.5)	69 (40.8)	0.688
Hypertension, *n* (%)	60 (52.2)	100 (59.2)	0.243
Hypercholesterolaemia, *n* (%)	51 (44.4)	83 (49.7)	0.376
Diabetes mellitus, *n* (%)	11 (9.5)	33 (19.5)	0.021
Smoking			
Never	51 (44.0)	77 (45.6)	0.960
Ex-smoker	54 (46.6)	77 (45.6)	
Current smoker	11 (9.5)	15 (8.9)	
Heart failure, *n* (%)	4 (3.5)	9 (5.4)	0.569
AF prior to study entry, *n* (%)	28 (23.9)	61 (36.3)	0.027
Educational age (years), mean (SD)	16.8 (3.5)	16.0 (2.5)	0.0288
NIHSS, median (IQR)	3.5 (2–9)	5.5 (2–11)	0.0210
Acute MoCA score, median (IQR)	25 (21–27)	23 (18–26)	0.0091
Discharge mRS, median (IQR)	1 (0–2)	1 (1–3)	0.0321
Further intracerebral event within 12 months of study entry, *n* (%)	6 (5.1)	9 (5.3)	0.941

Percentage values were calculated using the total number of patients for whom data were available as the denominator. *p* values are from independent t-tests (age, educational age, discharge mRS), Mann–Whitney *U* test (NIHSS, acute MoCA score), Fisher’s exact test (heart failure) or Chi-squared tests (remainder)

*AF* Atrial fibrillation, *IQR* interquartile range, *MoCA* Montreal cognitive assessment, *mRS* modified Rankin scale, *NIHSS* National Institutes of Health Stroke Scale, *SD* standard deviation

### Comparison of acute and 12 months MoCA performance

**Fig. 2:**
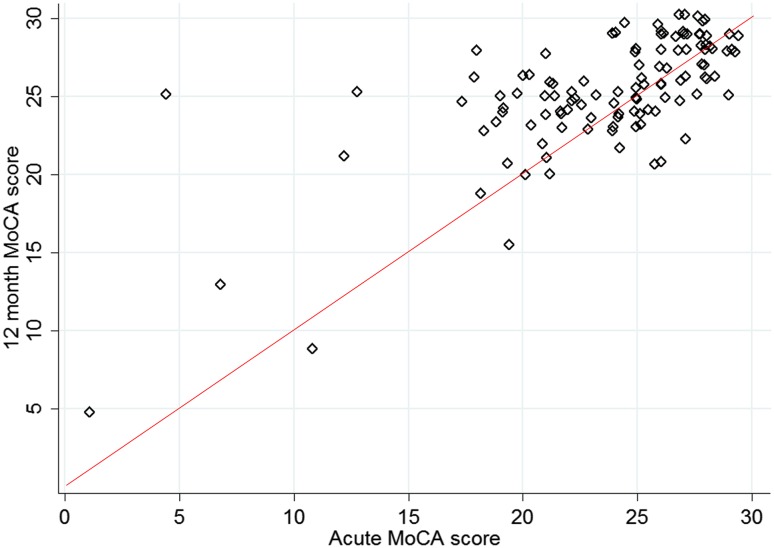
Distribution of acute and 12 months MoCA scores. Each patient is shown by a single diamond; the data have been jittered to show individual points. The line of equality is shown in red

Acute MoCA data were available for 114 patients with 12 month MoCA data (Fig. 2). The median time to acute MoCA assessment was 4 days (IQR 2–8 days). Overall, there was an improvement at 12 months (mean difference 1.69 points where maximum score is 30, *p* < 0.00001; Table 2). Scores improved across all subdomains except for attention (which showed a deterioration) and were statistically significant for visuo-executive function (mean difference 0.23 points, *p* = 0.0470), abstraction (mean difference 0.14 points, *p* = 0.0176) and delayed recall (mean difference 0.62 points, *p* = 0.0002).

**Table 2:** Comparison of MoCA performance acutely (median 4 days following ischaemic event) and at 12 months

	Comparison of means					Comparison of proportions		
	Maximum achievable score	Acute MoCA (*n* = 114); mean score (SD)	12 month MoCA (*n* = 117); mean score (SD)	Mean difference (95% CI)	*p* value	Acute MoCA (*n* = 114); impaired, *n* (%)	12 month MoCA (*n* = 117), impaired, *n* (%)	*p* value
Total score	30	23.55 (4.95)	25.25 (3.88)	1.69 (1.03–2.36)	< 0.00001	66 (57.9)	60 (51.3)	0.0719
Visuo-executive	5	3.77 (1.38)	4.00 (1.13)	0.23 (0.00–0.45)	0.0470	72 (63.2)	69 (59.0)	0.2888
Naming	3	2.75 (0.61)	2.82 (0.49)	0.07 (− 0.05 to 0.19)	0.2399	22 (19.3)	17 (14.5)	0.3173
Attention	6	4.98 (1.49)	4.70 (1.60)	− 0.28 (− 0.05 to 0.61)	0.0964	61 (53.5)	54 (46.2)	0.1228
Orientation	6	5.58 (1.06)	5.71 (0.73)	0.13 (− 0.05 to 0.32)	0.1628	25 (21.9)	24 (20.5)	0.5050
Language	3	2.11 (0.91)	2.25 (0.96)	0.13 (− 0.05 to 0.32)	0.1591	68 (59.7)	57 (48.7)	0.0269
Abstraction	2	1.58 (0.68)	1.72 (0.59)	0.14 (0.02–0.26)	0.0176	36 (31.6)	26 (22.2)	0.0233
Delayed recall	5	2.34 (1.62)	2.96 (1.53)	0.62 (0.31–0.94)	0.0002	102 (89.5)	98 (83.8)	0.1444

Impairment was defined as scoring less than full marks in a given domain; MoCA impairment was defined as previously (score < 26). Percentage values were calculated using the total number of patients for whom data were available as the denominator. For the comparison of mean scores, *p* values are from paired *t* tests, and for the comparison of proportion impaired, McNemar’s test was used

*CI* Confidence intervals, *MoCA* Montreal Cognitive Assessment, *SD* standard deviation

We also considered whether the proportion of participants impaired across domains (defined as scoring less than full marks) changed with time (Table 2). Fewer patients demonstrated MoCA impairments at 12 months (51.3% vs 57.9%, *p* = 0.0719), and there were lower proportions of impaired participants across all domains; this was statistically significant for language (48.7% vs 59.7%, *p* = 0.0269), and abstraction (22.2% vs 31.6%, *p* = 0.0233).

### Clinical and imaging associations of cognitive impairment 12 months following index ischaemic event

In our cohort, 51.3% (*n* = 60) had an abnormal MoCA score (< 26) at 12 months. When comparing those with and without MoCA-defined cognitive impairment at 12 months, those with impairment were older (mean age 75.6 years vs 70.5 years, *p* = 0.0022), had fewer years of education (mean 15.9 years vs 17.7 years, *p* = 0.0077), had a higher admission NIHSS (median score 5.5 vs 2, *p* = 0.0060), lower acute MoCA score (median 22 vs 27, *p* < 0.00001) and higher discharge mRS (median score 2 vs 1, *p* = 0.0004). In a multivariable logistic regression analysis including these variables, only acute MoCA score remained associated with MoCA impairment at 12 months (per-point increase, OR 0.73, 95% CI 0.59–0.91, *p* = 0.005).

Patients with cognitive impairment at 12 months had higher grades of pvWMH (IQR 0–1 vs 0–0, *p* = 0.0545) and had higher CAA scores (median score 0.5 vs 0, *p* = 0.0005). There were no statistically significant differences between the two groups in the imaging features of the index ischaemic lesion (presence of acute DWI lesion at study entry, side of index lesion, presence of multiple index lesions on DWI, presence of a cortical lesion, evidence of haemorrhagic transformation). In adjusted analyses (adjusted for age, educational age, discharge mRS and acute MoCA score), cognitive impairment at 12 months remained associated with CAA score (per-point increase, OR 4.09, 95% CI 1.36–12.33, *p* = 0.012) but not pvWMH grade (OR 1.15, 95% CI 0.54–2.44, *p* = 0.725).

### MoCA trajectory

**Fig. 3:**
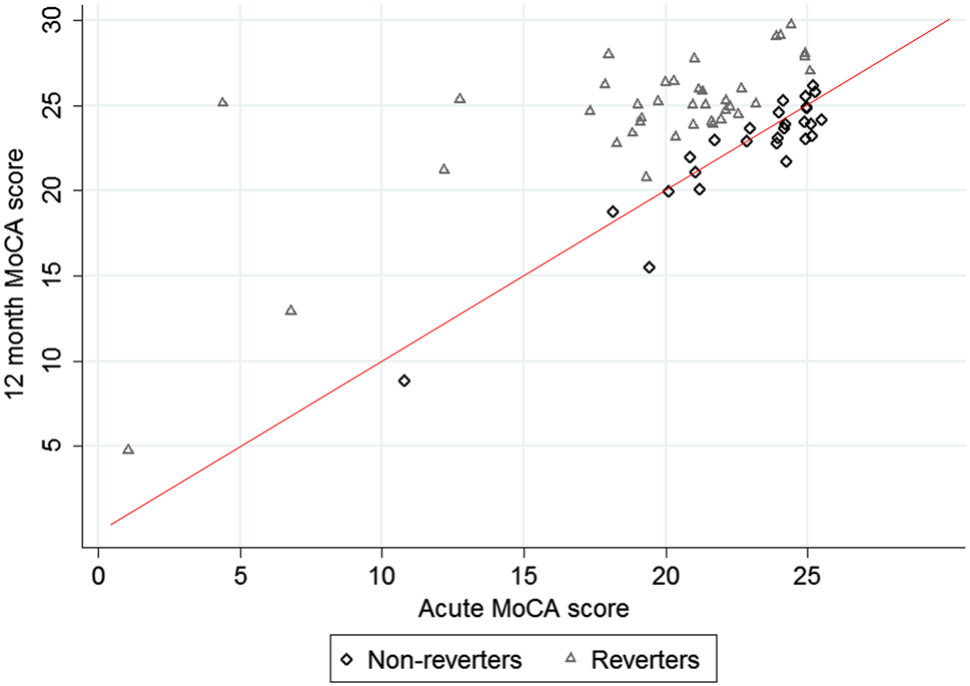
Distribution of acute and 12 months MoCA scores for reverters and non-reverters. Each patient is shown by a single symbol, as indicated by the key; the data have been jittered to show individual points. The line of equality is shown in red

In this cohort, 66 patients had an acute MoCA score below 26; of these, 59.1% (*n* = 39) were “reverters” (Fig. 3). Non-reverters had higher acute MoCA scores (median 24 vs 21, *p* = 0.0002) and lower 12-month MoCA scores (median 23 vs 25, *p* = 0.0008); there were no other clinical or demographic (including years of education) differences between the two groups.

The imaging characteristics of reverters and non-reverters are shown in Table 3. Non-reverters had lower baseline pvWMH grade (IQR 0–0 vs 0–1, *p* = 0.0752), but higher CSO-PVS grade (median grade 2 vs 1, *p* = 0.0306), and were more likely to have cerebral microbleeds (22.2% vs 2.6%, *p* = 0.016) and in particular, strictly lobar microbleeds (14.8% vs 0.0%, *p* = 0.024). Non-reverters also had a higher composite SVD (mean 0.88 vs 0.27, *p* = 0.0046) and CAA (mean 0.80 vs 0.25, *p* = 0.0007) scores. In unadjusted logistic regression analyses (Table 4), non-reversion remained positively associated with CSO-PVS grade (per-grade increase, OR 1.83, *p* = 0.029), cerebral microbleed presence (OR 10.86, *p* = 0.032), SVD score (per-point increase, OR 2.91, *p* = 0.015) and CAA score (per-point increase, OR 6.71, *p* = 0.001), and negatively associated with the presence of multiple lesions at study entry (OR 0.11, *p* = 0.040). Similar associations were observed in analyses adjusted for MoCA score (Table 4).

**Table 3:** Comparison of imaging characteristics of reverters vs non-reverters

	Reverters	Non-reverters	*p* value
*n* (%)	39 (59.1)	27 (40.9)	–
Structural imaging markers at study entry			
Imaging evidence of previous cortical infarct, *n* (%)	3 (7.7)	4 (14.8)	0.432
Lacunes, presence, *n* (%)	5 (12.8)	5 (20.8)	0.485
pvWMH grade, median (IQR)	0 (0–1)	0 (0–0)	0.0752
dWMH grade, median (IQR)	1 (0–1)	0 (0–1)	0.3217
CSO-PVS grade, median (IQR)	1 (1–2)	2 (1–3)	0.0306
BG-PVS grade, median (IQR)	1 (1–1)	1 (1–1)	0.1221
MTA grade, median (IQR)	1 (0–1)	1 (0–1)	0.7561
GCA grade, median (IQR)	1 (0–1)	1 (0–1)	0.9943
CMB, presence, *n* (%)	1 (2.6)	6 (22.2)	0.016
Strictly lobar CMB, *n* (%)	0 (0.0)	4 (14.8)	0.024
Composite SVD score, median (IQR)			
Median (IQR)	0 (0–0)	1 (0–1)	0.0046
Mean (SD)	0.27 (0.61)	0.88 (0.93)	
Composite CAA score, median (IQR)			
Median (IQR)	0 (0–0.5)	1 (0–1)	0.0007
Mean (SD)	0.25 (0.44)	0.80 (0.62)	
Imaging features of index ischaemic event			
Acute DWI lesion at study entry, *n* (%)	32 (82.1)	19 (76.0)	0.557
Side of index lesion, *n* (%)			
Left	11 (35.5)	6 (31.6)	0.249
Right	16 (51.6)	13 (68.4)	
Bilateral	4 (12.9)	0 (0.0)	
Presence of multiple lesions, *n* (%)	11 (34.4)	1 (5.3)	0.020
Cortical lesion, *n* (%)	21 (65.6)	14 (73.7)	0.756
Evidence of haemorrhagic transformation, *n* (%)	8 (20.5)	2 (8.7)	0.298

Percentage values were calculated using the total number of patients for whom data werer available as the denominator. *p* values are from Mann–Whitney *U* tests (where median and IQR are given), Chi-squared tests (acute DWI lesion at study entry) or Fisher’s exact test (remainder)

*BG-PVS* MRI-visible perivascular spaces in the basal ganglia, *CMB* cerebral microbleed, *CSO* MRI-visible perivascular spaces in the centrum semi-ovale, *DWI* diffusion-weighted imaging, *dWMH* deep white-matter hyperintensities, *GCA* global cortical atrophy, *IQR* interquartile range, *MoCA* Montreal cognitive assessment, *MTA* medial temporal atrophy, *pvWVH* periventricular hyperintensities, *SD* standard deviation

**Table 4:** Unadjusted and adjusted logistic regression analyses for predictors of non-reversion

	Unadjusted		Adjusted for acute MoCA score	
	OR (95% CI)	*p* value	OR (95% CI)	*p* value
pvWMH (per-grade increase)	0.41 (0.12–1.35)	0.143	0.50 (0.15–1.68)	0.264
CSO-PVS (per-grade increase)	1.83 (1.06–3.15)	0.029	1.96 (1.05–3.66)	0.035
BG-PVS (per-grade increase)	2.60 (0.74–9.19)	0.137	2.26 (0.63–8.02)	0.209
CMB (presence)	10.86 (1.22–96.34)	0.032	9.36 (0.92–95.34)	0.059
SVD score (per-point increase)	2.91 (1.23–6.88)	0.015	2.47 (1.02–6.00)	0.046
CAA score (per-point increase)	6.71 (2.10–21.50)	0.001	6.70 (1.88–23.98)	0.003
Presence of multiple lesions at study entry	0.11 (0.02–0.90)	0.040	0.11 (0.01–1.01)	0.051

Each model considered one imaging marker at a time

*BG-PVS* MRI-visible perivascular spaces in the centrum semi-ovale, *CAA* cerebral amyloid angiopathy, *CI* confidence interval, *CMB* cerebral microbleed, *CSO-PVS* MRI-visible perivascular spaces in the centrum semi-ovale, *MoCA* Montreal cognitive assessment, *pvWMH* periventricular hyperintensities, *SVD* small-vessel disease

## Discussion

MoCA-defined cognitive impairment at 12 months is common, observed in approximately half of our cohort, and associated with factors relating to brain resilience (age, educational age) and stroke severity (acute MoCA score, NIHSS, discharge mRS), as well as increases in a composite CAA score. Overall, we found that MoCA performance at 12 months improves compared with acute performance, and we show that the presence of structural imaging markers of small-vessel disease (CSO-PVS, cerebral microbleeds, composite SVD and CAA scores) is associated with non-reversion.

Our use of composite scores for the two most common small-vessel diseases provides new perspectives on the small-vessel mechanisms which might underlie post-stroke dementia. Composite scores are hypothesised to better reflect overall pathological burden and have shown associations with a number of clinical measures including cognitive performance [8, 18, 35, 36, 39]. In our study, we observed an association of 12-month cognitive performance with the composite CAA score. This score includes non-haemorrhagic markers of CAA such as CSO-PVS and WMH which do not feature in the current diagnostic criteria for CAA [21], and which might have more relevance in non-haemorrhagic patient populations. The association of CAA with dementia following intracerebral haemorrhage has been described [25], as have associations between strictly lobar microbleeds and executive function in patients with ischaemic stroke or TIA [14]. Whilst our finding that CAA score is associated with 12-month MoCA impairment should be interpreted with caution, given the low prevalence of haemorrhagic markers and the relatively small size of the cohort, it might provide further evidence that imaging markers of CAA are associated with cognitive performance beyond intracerebral haemorrhage, a finding already observed in non-haemorrhagic memory clinic populations [33, 42].

Our observation that overall cognitive performance can improve with time following an ischaemic event is in keeping with data from previous studies [2, 10, 17, 19, 29], as are the significant domain specific improvements in visuo-executive function, abstraction and delayed recall [27, 34]. As well as improvements in raw scores, we also found that levels of impairment were lower at 12 months for most domains. However, we did note that for a number of domains, whilst raw scores improved significantly, patients remained impaired range (as we defined it). This highlights the difficulties in quantifying deficits when considering individual domains.

We also describe the characteristics of patients with impaired acute performance who demonstrate an improvement of two or more points—so-called “reverters”[27, 34]—and those who do not. Whilst use of the term “reverter” has been criticised for suggesting that cognitive performance returns to normal [27], it is useful as a standardised method for defining improvement. Whilst we did not find any clinical or demographic differences between reverters and non-reverters (except for those relating to MoCA scores), there were imaging differences. Non-reverters appeared to have more evidence of small-vessel disease (CSO-PVS, cerebral microbleeds, and higher composite SVD and CAA scores), and were less likely to have had multiple acute DWI lesions at study entry. As discussed above, the association between both multiple lesions and lower acute MoCA scores amongst the reverters might suggest that multiple lesions are more likely to result in an acute reversible cognitive impairment—although the acute disturbance in this cohort does not seem to be typical for delirium, given the lack of attentional improvement with time. Replication of this work in larger cohorts will be important for confirming and better quantifying these observations.

The strengths of this study are its multicentre prospective design, and the detailed clinical and radiological descriptions available for the study participants. However, there are also some limitations. Firstly, only a subset of centres collected 12-month MoCA data, and even within these centres MoCA data were not collected for all potentially eligible participants. The excluded patients had more comorbidities, lower educational age, more severe ischaemic events (as defined by NIHSS and discharge mRS) and had lower acute MoCA scores, all of which are associated with poorer cognitive outcome at 12 months. Given this, and the fact that our cohort tended to have milder strokes (median NIHSS 3.5), the cognitive performance of our cohort might be better than expected and not representative of all cardioembolic ischaemic stroke or TIA cohorts. Additionally, we were unable to account for patients who were aphasic; this should be considered in the interpretation of our results. We note that group sizes for some analyses are small and the prevalence of haemorrhagic markers in our cohort was low; in view of this, these results should be interpreted cautiously. Our modest group sizes also precluded further subdivision into the reverter and non-reverter groups; in particular, a comparison of non-reverters with stable cognition vs those demonstrating further decline would be of interest, and an important area for future work. Cognitive assessments acutely after an ischaemic event period can be difficult to interpret, as cognitive performance can be influenced by intercurrent illness (such as coexisting infection, as one example) or complications relating to the ischaemic event itself (for example, seizures). Whilst we acknowledge that a slightly delayed assessment (for example, 2–4 weeks after an ischaemic event), might avoid these complicating factors, in routine clinical practice the first measurement of cognitive performance (using the MoCA and other tools) is often in the acute period, as in our study; the implications of early assessments are therefore of relevance. Finally, we only used a single measure (the MoCA) to estimate cognitive performance at a single time point; the MoCA has some intrinsic limitations, including the fact that it is primarily a screening tool and thus likely underestimates the severity and breadth of cognitive impairment that result from stroke [3, 4]. The absence of data on MoCA performance at additional time points, both before 1 year (for example, at 3 or 6 months) and beyond this (for example, at 2 years), is a further limitation; this would allow us to better define post-event cognitive trajectory, and the lack of assessment at additional time points is a limitation of our study design. Nevertheless, the positive findings and associations found show some promise that it might be a useful tool for monitoring for change over time.

We conclude that MoCA-defined cognitive impairment 12 months following an ischaemic event is common, and that structural imaging features of small-vessel disease appear associated both with deficits and a lack of improvement at 12 months. Further work that clarifies the role of small-vessel diseases in this context will be essential for refining future cognitive rehabilitation strategies.
